# Comparing Intravenous Insertion Instructional Methods with Haptic Simulators

**DOI:** 10.1155/2017/4685157

**Published:** 2017-01-29

**Authors:** Lenora A. McWilliams, Ann Malecha

**Affiliations:** ^1^University of Houston, 14000 University Blvd., Sugar Land, TX 77479, USA; ^2^Texas Woman's University, 6700 Fannin Street, Houston, TX 77030-2343, USA

## Abstract

*Objective*. The objective of this review was to compare traditional intravenous (IV) insertion instructional methods with the use of haptic IV simulators.* Design*. An integrative research design was used to analyze the current literature.* Data Sources*. A search was conducted using key words intravenous (IV) insertion or cannulation or venipuncture and simulation from 2000 to 2015 in the English language. The databases included Academic Search Complete, CINAHL Complete, Education Resource Information Center, and Medline.* Review Methods*. Whittemore and Knafl's (2005) strategies were used to critique the articles for themes and similarities.* Results*. Comparisons of outcomes between traditional IV instructional methods and the use of haptic IV simulators continue to show various results. Positive results indicate that the use of the haptic IV simulator decreases both band constriction and total procedure time. While students are satisfied with practicing on the haptic simulators, they still desire faculty involvement.* Conclusion*. Combining the haptic IV simulator with practical experience on the IV arm may be the best practice for learning IV insertion. Research employing active learning strategies while using a haptic IV simulator during the learning process may reduce cost and faculty time.

## 1. Introduction

One of the most commonly performed basic nursing skills is intravenous (IV) catheter insertion. IV catheterization, cannulation, or insertion is a complex [1] and invasive procedure [2]. While it is the most common and important skill performed in the clinical setting, it is also a technically difficult procedure [3]. Reinhardt et al. [4] note that learning how to insert an IV catheter is the most challenging skill taught in nursing school. Traditional methods of instruction for IV insertion vary between nursing and medical students. The nursing student IV education typically consists of faculty didactic presentation followed by faculty demonstrating the procedure on an IV arm task trainer. For this teaching method, students practice under faculty supervision followed by a skills competency check off assessment. The traditional teaching method for medical students varies and may consist of didactic instruction followed by either practicing on a simulated arm, practicing the actual procedure on students or patients [5], learning by doing [6], or see-one do-one format [7].

Traditional methods of teaching IV insertion can be time consuming and costly [8]. Sotto et al. [7] state that not only the current methods of teaching IV insertion are cost ineffective but also opportunities for practicing the procedure are inconsistent, with limited variability, and generally delegated to ancillary personnel such as lab assistants. Engum et al. [6] discuss the importance of practitioners developing clinical skills for invasive procedures prior to working with patients. Students need to master high risk skills, such as IV catheter insertion, prior to performance in the clinical setting. Proficiency of IV insertion may prevent serious patient complications including infiltration, phlebitis, or pain [9].

The use of patient simulators in education allows students to learn and experience real world situations, while ensuring that patients receive safe, competent treatment as well as reducing cost of instruction and practice [8]. The use of simulators provides opportunities for students to practice and perfect skills in a safe nonthreatening environment [10] while making errors without harm or discomfort to a patient [6]. During simulation, students become active learners [10]. They construct new knowledge by attaching meaning to current or past experiences, which is then assimilated for future encounters [11]. More and more, the skill of IV insertion is being taught with a haptic IV simulator. This device includes a catheter/hub assembly and an interface that allows students to palpate a vein, stretch the skin, and feel resistance during venipuncture. Additionally, during the simulated vein cannulation, a computer screen provides immediate feedback related to bleeding, bruises, and swelling.

Nurse educators must prepare nursing students to be competent as they transition from student to clinical practitioner [12]. Nursing students need opportunities to practice and learn how to perform safe and competent IV insertion or cannulation as inappropriate cannulation may have harmful effects, including infiltrations, phlebitis, and pain [13]. With the increasing use of simulation in nursing education, it is unclear if traditional methods of teaching IV insertion or the use of an IV simulator result in better skill performance competency.

## 2. Aim

An integrated literature review was performed to examine and/or compare traditional IV insertion instructional methods with haptic IV simulators and compare the outcomes of the instructional methods.

## 3. Search Methods and Strategy

A search of the databases Academic Search Complete, CINAHL Complete, Education Resource Information Center and Medline was conducted in order to locate published articles on methods and simulators used to teach IV insertion or cannulation. The search was limited to English language articles published between the years 2000 to 2015. Search terms employed were a combination of intravenous (IV) insertion, cannulation, or venipuncture and simulation. Of the 51 articles retrieved, 11 met the inclusion criteria for research studies utilizing haptic IV simulators for the purpose of teaching IV insertion or cannulation. Whittemore and Knafl's [14] data analysis strategies were used to provide structure and rigor while employing a constant comparison approach to the literature review process. For this integrative review, these strategies included reducing data into subcategories, organizing data using a matrix format, followed by comparison, analysis, and verification of overarching themes and conclusions of the subcategories. Synthesis of these subcategories was then integrated to provide a comprehensive summation of the topic of IV instructional methods using a simulator.

## 4. Results

### 4.1. Comparison of Instructional Methods and Performance

#### 4.1.1. Setting and Sample

Table 1 summarizes the IV simulators and instructional methods for this review. Of the 11 articles reviewed, 6 studies were conducted in the United States while the other 5 studies were conducted in Greece, Hong Kong, Korea, Philippines, and Sweden. The participants varied from nursing students (*n *= 5 studies), to medical students (*n *= 2 studies), to registered nurses (*n *= 2 studies). One study utilized both nursing and medical students while another one used a combination of medical students, graduate medical doctors, and nurses who were experts in IV cannulation.

#### 4.1.2. Teaching and Practice

Almost every study (*n* = 10) compared the use of a haptic IV simulator to more traditional teaching methods. The haptic IV simulators used in these studies included the CathSim Intravenous Training System (CS) from Immersion Medical, Inc., Laerdal's Virtual IV (VIV) training system, and the virtual reality/haptic IV training simulator from the Republic of Korea [3]. The majority of the instructional methods followed a pattern of (a) pretest-lecture-practice-performance testing or (b) lecture-pretest-practice-performance testing. Traditional instruction included students practicing on a plastic IV arm (*n *= 8 studies), on each other (*n* = 1), on a healthy volunteer (*n* = 1), or on a manikin (*n* = 1).

#### 4.1.3. Skill Performance

In eight of the eleven studies, the students' skill performance was evaluated via actual IV insertion attempt on a patient [3, 4, 7, 10, 13] or a human volunteer [6, 8, 15]. For three studies, skill performance was evaluated with the use of an IV arm [5], on a haptic IV simulator [16], or a combination of an IV arm and a haptic simulator [9].

In six studies, the use of a haptic IV simulator or a combination of an IV arm and a haptic IV simulator showed improvement in performance skills. Bowyer et al. [5] indicated that while all groups improved on performance as evaluated by the faculty, the group using the VIV simulator showed greater improvement over the IV arm group. They concluded that both haptic IV simulators (VIV and CS) were at least equal to the traditional method of teaching IV insertion, which is costlier and faculty intensive. Jamison et al. [9] noted that skill performance was moderately related to improved cognitive posttest scores for the CS group.

In three of the eleven studies, students' IV cannulation success rates were compared and no differences were noted between the traditional training method and training on the haptic IV simulator [4, 6, 15]. Chang et al. [13] were unable to determine if one training method was superior to another due to confounding factors and unequal group composition, even though both groups had successful cannulation rates.

However, Sotto et al. [7] found that the group using the VIV simulator had a greater success rate in starting an IV on a patient and lower band constriction and total procedure time than the traditional method of teaching IV insertion. Wilfong et al. [3] noted that the VIV simulator group required fewer attempts in order to be successful at inserting an IV on a patient compared to the group who practiced on each other. Jung et al. [8] noted that students' procedure scores were higher if they trained on both the IV arm and haptic IV simulator and task time was shorter than those students using only the haptic IV simulator. Loukas and colleagues [16] compared outcomes between groups using the VIV simulator. The groups consisted of novice students (no IV experience) or intermediate graduates (less than ten IV insertions) and the outcomes measured included learning curve, errors, and task time on the VIV simulator. After training on the VIV simulator, both the novice and intermediate groups' scores for the outcomes measured were similar to those of a group of experts who were proficient in IV insertion (*p *> .1).

#### 4.1.4. Knowledge, Satisfaction, and Students' Expectations

Engum et al. [6] study results indicated that students' posttest scores and satisfaction scores were higher for the traditional methods group. In addition, both nursing and medical students preferred the traditional method of one-to-one faculty-student instruction over the CS system. Feedback from the traditional group indicated that they enjoyed working with faculty and touching the equipment but wanted more practice time in smaller groups with more instructors per group. Feedback from the CS group indicated that while the haptic IV simulator was not “real world,” they enjoyed the simulator's variety of case scenarios, the instant feedback feature, and the ability to self-pace their own learning without harm to a patient. Overall, medical students were more accurate with IV placement but were more businesslike and task driven while the nursing students were more emotionally involved and concerned for their patients.

Two studies showed that the haptic groups' posttest knowledge scores improved [9] and assessment tool scores were greater [7] compared to the traditional groups. Reyes et al. [15] indicated that both groups (traditional and VIV) made cognitive gains. After posttesting [15], the traditional group was given the opportunity to train on the VIV simulator. Student satisfaction with the VIV experience showed that the group who initially trained on the VIV felt that the haptic IV simulator helped with clinical training (64% versus 42%), was time well spent (57% versus 50%), and helped develop clinical skills (35% versus 32%) compared to the group who received training on the VIV after their traditional method training. The traditional method group felt that their VIV experience helped test decision making (57% versus 50%) and challenged critical thinking (41% versus 21%) compared to the original VIV simulation group. Both groups recommended that VIV simulation be continued (VIV group 79%; traditional group 66%).

Reyes et al. [15] concluded that IV performance was reinforced by practicing using the VIV simulator. Jung et al. [8] noted that repeated practice reduced students' anxiety while Chang et al. [13] found no differences in state anxiety levels between groups. Jung et al. [8] indicated that students who used both the IV arm and the haptic IV simulator were more satisfied with learning the procedure and that by combining both methods students were able to learn the skill in a timely manner using fewer consumables.

In the Johannesson et al. [10] study, students using a haptic simulator were asked an open-ended question regarding their learning expectations before training, after training, and after skill examination. Pretraining expectations included learning the practical technique, gaining confidence and insight, meeting the patient, and managing the situation. Posttraining findings noted that the simulator was helpful in building confidence and was a valuable learning tool. However, postskill examination results found the simulator was less valued as a learning tool. Overall, students' learning expectations were not met. Students felt that the haptic IV simulator did not equate with reality, that it limited their experience from a holistic perspective, and that the experience did not provide opportunity to practice communication skills. However, they expressed appreciation for the simulators' realism, variations in case scenarios, value in giving feedback, and opportunity for repeated practice. Students indicated that their awareness of patients' conditions and their effect on the patient's veins increased and they become more confident in their skills. The authors concluded that the haptic IV simulator was useful in the learning process when combined with the IV arm and that repeated practice built confidence in a safe environment while using active and independent learning. Loukas et al. [16] also noted that the haptic IV simulator was useful for learning the principles of IV cannulation and both Loukas et al. [16] and Wilfong et al. [3] noted that the haptic IV simulator increased self-confidence.

### 4.2. Cost

Five articles in this review expressed concern regarding the cost of either the haptic IV simulator technology or the traditional approach (faculty/lab time intensive plus supplies) when teaching IV insertion. As of July 2015, the approximate cost of one haptic IV simulator ranges between US$20,000–US$25,000. Jung et al. [8] note that more economical training methods are needed in order to prepare practitioners who are skilled using learning methods that are effective.

Wilfong et al. [3] identified the cost of the haptic IV simulator as being an issue in developing countries but also mentioned that the traditional method of teaching IV insertion requires a high faculty-to-student ratio which is also costly. Bowyer et al. [5] noted that haptic IV simulators are as effective in teaching IV insertion as the traditional method, which consists of didactic instruction followed by practice on a simulated arm or practicing the procedure on another student or patient. However, the traditional method is costlier in terms of supplies, equipment, and faculty time [5]. Reinhardt et al. [4] addressed the cost of the haptic IV simulator in comparison to the IV training arm while Engum et al. [6] noted the importance of keeping cost down in order to gain academic support. Jung et al. [8] stated that haptic IV simulators are cost effective as students do not need to use consumables during the learning process and they provide repeated educational opportunities for student practice. Jung et al. [8] recommended combining the haptic IV simulator with the IV arm to assist students with learning skills in a timely manner.

## 5. Discussion

Current use of haptic IV simulators and traditional training methods continue to have different outcomes. For this review, some of the studies indicated a decrease in student anxiety levels and an increase in cognitive gains with practice but these results were not related to the method of IV instruction. Other studies indicated performance improvement by a decrease in insertion time and band constriction time with the use of the haptic IV simulator.

Students' learning expectations related to the haptic IV simulator were high. They presumed that the technology was going to teach all aspects of IV insertion, including successful cannulation, when in reality the IV simulator teaches the process. Students continue to need faculty support and feedback during practice on the IV arm but during the learning process other options may be more feasible and cost effective. It is interesting to note that, even with the availability of IV plastic arms and haptic simulators, some educational programs still have students practicing on each other as well as on patients.

Intravenous catheter insertion is one of the most challenging skills taught in nursing school. A nurse's ability to insert an IV successfully depends on experience, number of insertions performed, and patient factors such as veins that roll or are resistant to venipuncture and color or turgor of the skin [17]. More importantly, the patient's perceptions of how caring the nurse is can be linked to his/hers beliefs about the nurse's skill in performing the procedure [17].

Simulation is an educational strategy that provides opportunities for students to practice skills in a risk-free environment without fear of harming patients [18]. Haptic IV simulators are useful in learning the process and skills training for IV insertion and cannulation [3, 5, 7, 9, 10, 15, 16]. Haptic IV simulators allow students to practice IV procedures repeatedly on complex patient scenarios [18] while developing proficiency and critical learning skills without using costly consumables and faculty time. While there is little information on the cost of the haptic IV simulators and virtually no information regarding the cost of consumables or faculty time needed to teach this skill, a cost comparison between these instructional methods would be beneficial.

Alexandrou et al. [19] concluded in their review of the literature on the training of vascular access for undergraduate clinicians that no method of training was found to be superior to another. However, the results of the current review lean towards more positive outcomes. The use of haptic IV simulators to teach the process of inserting an IV decreased the number of attempts needed to be successful when inserting an IV on patients [3] and reduced the total IV insertion procedure time and band constriction time [7, 16].

## 6. Conclusion

Based on this review and noted by Johannesson et al. [10] and Jung et al. [8] the best IV instructional method would combine the haptic IV simulator followed by practice time on the IV arm. During the learning process, group work while using the haptic IV simulator may be one alternative to decrease cost while providing support to group members. Only one of the studies in this review [10] trained 2 students together for one hour while using the CS followed by practice on an IV arm. Further research using cooperative learning or other active learning strategies while using the haptic IV simulator may be beneficial and could potentially reduce the overall cost of learning IV insertion.

## Figures and Tables

**Table 1:** IV simulators and instructional methods.

Author Year	Sample/setting	Purpose	Variables	Methods/analysis	Findings	Instructional methods
Bowyer et al. (2005) [5]	*N* = 34 3rd year medical students, Maryland, USA	Compare cannulation (skill) performance	INV^1^ 4 groups: Each other (EO) Virtual IV (VIV) CathSim (CS) IV arm (IVA) DV^2^ Skill performance	RCT; Pretest Posttest *t*-test ANOVA	All groups improved: EO: *p* < .0003 VIV: *p* < .0003 CS: *p* < .02 IVA: *p* < .009 All: *p* < .00001 VIV greater improvement than IVA group *p* < .026	5-minute training video Pretest Random assignment Practice: EO: 1 hour 2 students per faculty VIV: 1 hour alone CS: 1 hour alone IVA: 1 hour alone Posttest Skill performance on the IVA

Chang et al. (2002) [13]	*N* = 28 community nurses, Hong Kong	Compare outcomes between 2 instructional methods	INV 2 groups: IV Arm (IVA) CathSim (CS) DV Success rates Skill performance Anxiety	Quasi-experimental; Posttest *t*-test	Success rates on initial attempt: (%) IVA: 85.71 CS: 64.29 Successful cannulation rate: (%) IVA: 100 CS: 92.86 Skill performance: M (SD) IVA: 23.29 (1.54) CS: 22.86 (1.83) *p* = .509 Trait Anxiety Level: M (SD) IVA: 21.42 (1.69) CS: 21.14 (2.82) *p* = .418 State anxiety level: M (SD) IVA: 29.50 (5.00) CS: 28 (4.64) *p* = .749	Lecture Random assignment Practice: Supervised: 2 hours Independently: 1 week Posttests Skill performance on patient

Engum et al. (2003) [6]	*N* = 163 students, Indiana, USA *n* = 70 BS nursing students *n* = 93 3rd year medical students	Compare outcomes between 2 instructional methods	INV 2 groups: IV arm (IVA) CathSim (CS) DV Cognitive gains Student satisfaction Self-efficacy/reliance Documentation Patient feedback Skill performance	Quasi-experimental; pretest, posttest *t*-test *χ*^2^	*All students* IVA group higher scores: Cognitive gains: *p* = .013 Student satisfaction: *p* < .0001 Self-efficacy/reliance: *p* = .0146 Documentation: *p* = .014 Instruction helpful: *p* < .01 Skill performance not significant *Nursing student group* IVA group higher scores: Cognitive gains: *p* = .0064 Student satisfaction: *p* < .0002 Self-efficacy/reliance: *p* = .0167 *Medical student group* IVA group higher scores: Student satisfaction: *p* = .043	Pretest Only cognitive gains IVA group: Self-study module with video 90 min faculty instruction Practice CS group: Self-study module 90 min independent learning on CS Practice Posttest All DV Skill performance on volunteer

Jamison et al. (2006) [9]	*N* = 18 BS nursing students, Midwestern USA University	Compare outcomes between 2 instructional methods	INV 2 groups: IV arm (IVA) CathSim (CS) DV Knowledge Skill performance Educational practices Design features	Exploratory; pretest posttest *t*-test	*Knowledge* Only CS group improved: *p* < .05 *Skills performance* CS group knowledge related to skill performance: *p* < .05 *CS group* Most important educational practices were feedback and diverse ways of learning Most important simulation design features were feedback and cues	Lecture Pretest Knowledge Random assignment Both groups: Practice Skill performance on IVA or CS Posttests Knowledge Educational practices Design features

Johannesson et al. (2010) [10]	*N* = 24 BS nursing students, University of Linkoping, Sweden	Investigate students' learning expectations before and after training related to IV catheterization	3 measurement times: Pretraining Posttraining Postexam DV Learning expectations (pretraining) Fulfillment of expectations (posttraining and postexam) Curricular goal expectations (pretraining and postexam) Skill examination	Descriptive; pretest posttest Wilcoxon signed rank test Open-ended questions	Pretraining learning expectations: 20/21 looked forward to using CS 19/21 looked forward to using IVA Fulfillment of expectations was met after training: Learning support from teacher: *p* = .038 17/22 CS was valuable learning tool Fulfillment of expectations after exam: 4/20 CS was valuable learning tool Learning support from teacher: *p* = .038 Curricular goal expectations after exam: Decrease in ability to Organize actions: *p* < .001 Explain materials/procedure: *p* = .008 Interact with patient: *p* = .003 Be sensitive to patient: *p* = .004 8/20 felt prepared	Randomization Lecture Pretraining learning expectations Training: Introduction Practice in pairs on CS and then IVA Practice on own Posttraining Fulfillment of expectations Posttest: Skills examinations Curricular goal expectations Skill examination on patient

Jung et al. (2012) [8]	*N* = 114 nursing students, Korea	Compare outcomes between 3 instructional methods	INV: 3 groups: IV arm (IVA) IV Sim (IVS) IVA/IVS DV: Anxiety Skills performance Satisfaction Knowledge	RCT *t*-test Mann–Whitney *U* test One-way ANOVA	State-anxiety decreased IVA: *p* = .004 IVA/IVS: *p* = .002 VAS-anxiety decreased IVA: *p* = .012 IVS: *p* = .006 IVA/IVS: *p* < .001 *Skills performance* IVA/IVS group: Scores > than other groups: *p* = .015 Task time < than IVS: *p* = .007 *Satisfaction* Overall teaching effectiveness: IVA/IVS and IVA > IVS: *p* = .005 Learning procedure: IVA/IVS group most satisfied: *p* = .014	Random assignment Lecture and video Pretest anxiety Demonstration on a healthy volunteer Demonstration on training aid Practice Posttest anxiety Skills performance on volunteer

Loukas et al. (2011) [16]	*N* = 53 medical students and nurse experts, Athens, Greece	Compare outcomes between groups using VIV	INV: 3 groups: Novice (N) Intermediate (I) Experts (E) DV: Performance on VIV: Learning Curve Procedure Time Errors Efficacy Confidence	Quantitative and qualitative mixed method Friedman, Kruskal-Wallis, Kuder-Richardson tests	*Performance* Learning curve plateaus after N = 23 attempts I = 15 attempts Procedure time decreased *p* < .01 Errors decreased: Critical: *p* < .01 Noncritical: *p* < .05 Posttraining performance N = I = E: *p* > .01 *Efficacy* Learning principles: *p* < .05 Realism and content: *p* < .05 *Confidence* Increased: *p* < .05	Pretest: 3 scenarios on VIV Intervention: Groups N and I: Lecture Practice 9 different scenarios Posttest: 3 scenarios on VIV

Reinhardt et al. (2012) [4]	*N* = 94 BS nursing students, New Mexico, USA	Compare outcomes between 3 instructional methods and sequencing of methods	INV: 3 Groups: IV arm only (A) VIV then A (VA) A then VIV (AV) DV: Skill performance Confidence Clinical proficiency	RCT ANOVA *t*-test *χ*^2^ Pearson correlation	All groups were similar on skill and confidence scores. Clinical proficiency: A 87.5% VA and AV 84.2%	Random assignment Lecture Demonstration on A Practice Skills performance on patient

Reyes et al. (2008) [15]	*N* = 28 LPN student nurses, Washington, USA	Compare outcomes between 2 instructional methods	INV: 2 groups: Virtual IV (VIV) IV arm (IVA) DV: Cognitive gain Skill performance Student satisfaction	RCT *t*-test Fisher's Exact	Cognitive gain within groups: M (SD) VIV: 14.7 (11) IVA: 11.6 (11.26) *p* < .001 Skill performance: success rate on initial attempt (%): VIV: 64 IVA: 78 Student satisfaction: Recommend continued use of VIV (%) VIV 79 IVA 66	Random assignment Pretest Cognitive gain Review IV competency VIV: Orientation on VIV Practice IVA: Faculty instruction and demonstration Posttest Skills performance on volunteer IVA: VIV training opportunity IVA and VIV: Cognitive test Satisfaction survey⁡

Sotto et al. (2009) [7]	*N* = 40 medical students, Manila, Philippine	Compare outcomes between 2 instructional methods	INV: 2 groups: See-1Do-1 (S1D1) VIV DV: Success rate Skill performance Band constriction time Procedure time	Randomized posttest *t*-test *χ*^2^	Success rates (%): S1D1: 15 VIV: 40 *p* < .05 Skill performance score: M (SD) S1D1: 44 (15.3) VIV: 56 (19.2) *p* < .03 Band constriction time: M (SD) S1D1: 240 (38) VIV: 159 (31) *p* < .05 Procedure time: M (SD) S1D1: 380 (48) VIV: 277 (53) *p* < .05	Instructional video on VIV Stratified random assignment S1D1: Demonstration on a patient VIV: Practice until successful Posttest Skill performance on patient

Wilfong et al. (2011) [3]	*N* = 46 registered nurses, Pennsylvania, USA	Compare outcomes between 2 instructional methods	INV: 2 groups: VIV and patient manikin (VIVPM) Each other (EO) DV: Skill performance Success Rate Complication	RCT *t*-test Mann–Whitney *U* test *χ*^2^	Success rate on 1st attempt: VIVPM (median = 1.00) EO (median = 2.00): *U* = 143; *p* < .043 Complications on initial attempt (%) VIVPM: 21 EO: 33	Random assignment pretest Self-assessment VIVPM: Presentation Review policies and procedures Practice Perform IV on simulators EO: Presentation Review policies and procedures Practice on each other Post training: IV attempts on patients

^1^INV: independent variable.

^2^DV: dependent variable.
